# Prenatal sonographic diagnosis of fetal valproate syndrome: a case report

**DOI:** 10.1186/s13256-016-1094-1

**Published:** 2016-11-03

**Authors:** Norihiko Kikuchi, Satoshi Ohira, Ryoichi Asaka, Kyoko Tanaka, Akiko Takatsu, Tanri Shiozawa

**Affiliations:** Department of Obstetrics and Gynecology, Shinshu University School of Medicine, 3-1-1 Asahi, Matsumoto, 390-8621 Japan

**Keywords:** Epilepsy, Fetal valproate syndrome, Prenatal diagnosis, Ultrasonography, Preconceptional counseling

## Abstract

**Background:**

Prenatal exposure of mother to valproate (VPA) causes teratogenic effects in the fetus, namely fetal valproate syndrome (FVS). We report a case of fetal valproate syndrome rarely diagnosed by prenatal sonographic examination.

**Case presentation:**

Our patient was a female infant who was born to a 27-year-old nulliparous Japanese woman with epilepsy. The mother was diagnosed with infantile epilepsy at 1 year of age and had been using three antiepileptic drugs, including valproate, but preconceptional counseling was not performed. At 25 weeks of gestation, contracture of the fetal right wrist joint suggestive of a radial ray defect was observed by transabdominal ultrasonography. The fetus demonstrated growth retardation starting from 32 weeks of gestation. In addition, saddle nose as a facial anomaly was detected by three-dimensional ultrasound at 37 weeks of gestation. Accordingly, we suspected that the fetus had fetal valproate syndrome. At 39 weeks of gestation, the mother delivered an infant weighing 2056 g. The neonate had characteristic features of fetal valproate syndrome, such as facial configuration, slight muscular hypotonia of the whole body, breathing problems, right-hand articular contracture accompanied by radial ray defect, and cardiovascular malformation.

**Conclusions:**

When obstetricians manage epileptic pregnant women without enough preconceptional counseling or adjustment for antiepileptic drugs, careful sonographic observation of the fetus is mandatory.

## Background

Valproate (VPA) is used for the treatment of epilepsy and mood disorders [1]. Prenatal exposure of the mother to VPA causes teratogenic effects in the fetus, namely fetal valproate syndrome (FVS). FVS is characterized by a number of abnormalities, including neural tube defects; congenital heart defects; limb defects; genitourinary defects; brain, eye, and respiratory anomalies; and abdominal wall defects [2]. Although FVS is well-known as a newborn disease, reports of prenatal ultrasonographic diagnosis are limited, and only two cases have been reported in the English-language literature. We report another case of FVS diagnosed by prenatal ultrasonography. Careful ultrasonographic observation might be useful for predicting FVS in pregnant women using antiepileptic drugs, including high-dose VPA, without enough preconceptional counseling.

## Case presentation

Our patient was a female infant who was born to a 27-year-old Japanese nulliparous woman. The mother had been diagnosed with infantile epilepsy at 1 year of age. Her antiepileptic therapy was discontinued at the age of 7 years because she had no symptoms, but she had an epileptic seizure at the age of 17 years, and treatment with the antiepileptic drugs was resumed. She married at the age of 25 years; however, she did not receive preconceptional consultation. Because she had slight mental retardation, she did not report her wish for a pregnancy to her physician. At the age of 27 years, she was referred to our hospital from a primary obstetrical clinic at 13 weeks of gestation. She was obese, with a body mass index of 30 kg/m^2^. The antiepileptic drugs she was taking included 1400 mg/day of VPA, 140 mg/day of phenobarbital (PB), and 1200 mg/day of carbamazepine (CBZ). The blood concentrations of the drugs were 80 μg/ml VPA (effective blood range is from 40 to 100 μg/ml), 26.1 μg/ml PB (10–30 μg/ml), and 10.5 μg/ml CBZ (4–12 μg/ml). Despite the mother’s use of three antiepileptic drugs, she had convulsions about three times per week. Therefore, reduction or alteration of the drugs was difficult. She was informed of her epileptic status and the teratogenic effects of VPA, but her wish to have a baby was so strong that she continued her pregnancy with oral folic acid of 5 mg/day.

**Fig. 1:**
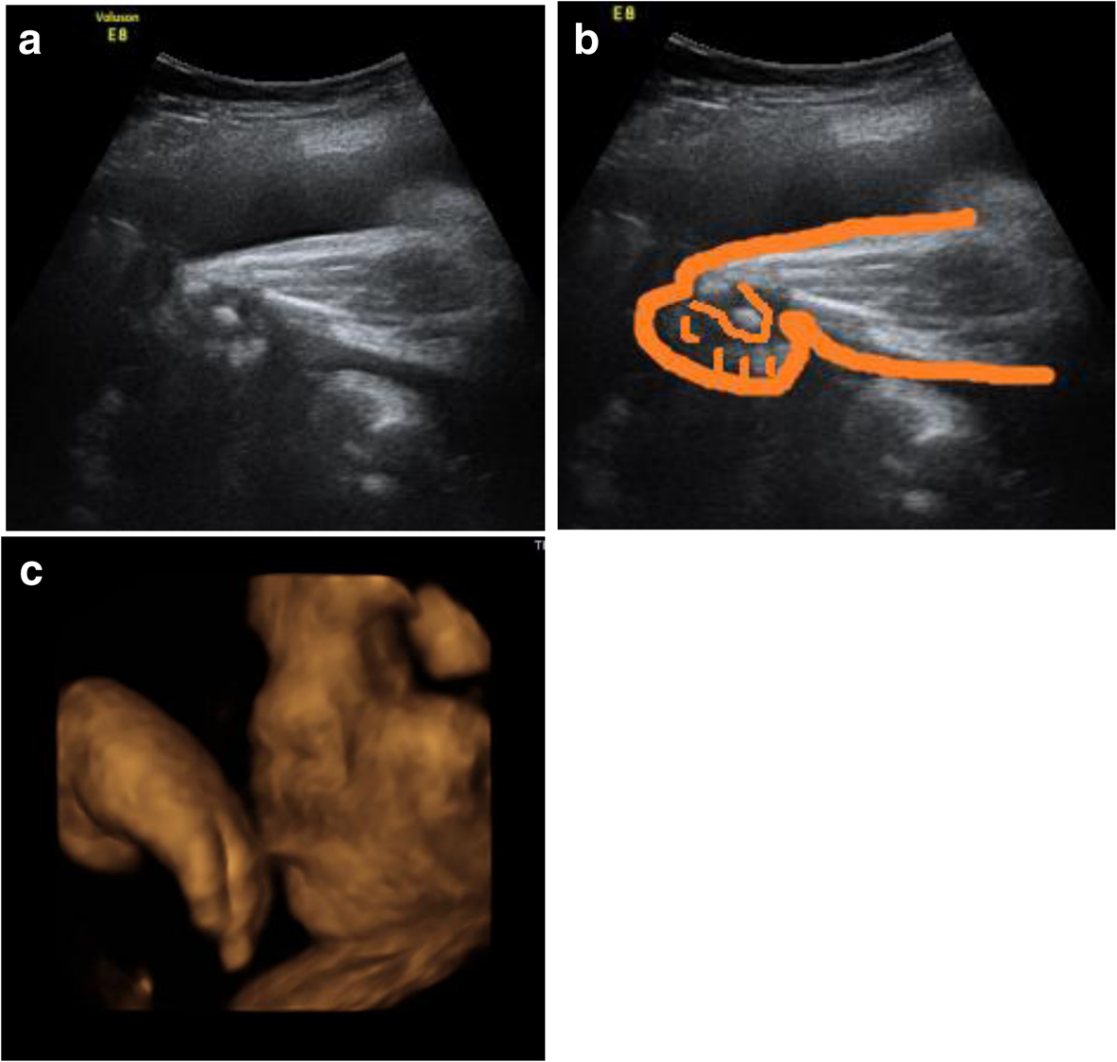
Prenatal ultrasonographic image. **a** Contracture of the fetal right wrist joint is observed at 25 weeks of gestation. **b** Schematic image of the right wrist joint. **c** Saddle nose as a facial anomaly is observed on this three-dimensional ultrasound obtained at 37 weeks of gestation

Although second-trimester transabdominal ultrasonography was performed at 20 weeks of gestation, fetal malformation was not detected because of a poor ultrasound image due to maternal obesity. At 25 weeks of gestation, contracture of the fetal right wrist joint suggestive of a radial ray defect was detected by ultrasonography (Fig. 1a, b). The fetus demonstrated growth retardation starting from 32 weeks of gestation. After that, fetal ultrasonography was performed weekly until delivery. At 34 weeks of gestation, the estimated fetal weight was 1660 g (−2.5 SD: standard deviation), the biparietal diameter was 74.9 mm (−2.8 SD), mother’s abdominal circumference was 261.7 mm (−1.5 SD), and the femur length was 58 mm (−1.3 SD). The right ulna was shorter than the left ulna; the right was 35 mm (−8.4 SD), and the left was 53 mm (−1.1 SD). The right radial ray was not detected. At 37 weeks of gestation, saddle nose as a facial anomaly was observed on a three-dimensional ultrasound (Fig. 1c). Therefore, right radial ray defect and saddle nose caused by FVS were suspected.

**Fig. 2:**
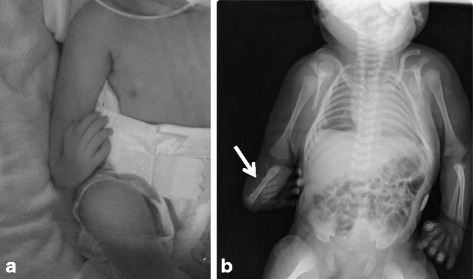
**a** The neonate’s physical examination reveal bend and contracture of right wrist joint. **b** A right radial ray defect is observed on this x-ray (*arrow*)

The serum levels of antiepileptic drugs were within effective ranges; however, multiple syncopes occurred. Fetal growth retardation was not improved, but growth arrest was not observed. The mother went into active labor at 39 weeks and 2 days of gestation and delivered a female infant weighing 2056 g with Apgar scores of 8 and 8 at 1 and 5 minutes, respectively. The neonate showed slight muscular hypotonia of the whole body and respiratory distress. The neonate’s physical examination revealed bend and contracture of the right wrist joint (Fig. 2a), right radial ray defect (Fig. 2b), a ventricular septal defect (VSD), an atrial septal defect (ASD), and a patent ductus arteriosus (PDA) in addition to characteristic facial configurations such as euryopia, broad nasal root, saddle nose, shallow philtrum, and low-set ears. The neonate’s features were compatible with FVS. PDA ligation was performed when she was 13 days old, and cast immobilization of the right forward arm was started when she was 89 days old. A follow-up examination at age 12 months demonstrated developmental and growth retardation.

## Discussion

The recommended treatment for a woman with epilepsy who wishes to have children is to use a single drug or newer antiepileptic drugs such as lamotrigine or levetiracetam [3–5]. Although avoidance of VPA is an option to prevent FVS, there are cases where the control of convulsions is impossible without VPA. There is a dose-effect relationship between fetal malformations and VPA exposure during the first trimester of pregnancy; that is, higher VPA doses are associated with a significantly greater risk than lower doses. Several investigators have reported a higher fetal malformation risk with maternal VPA doses above 1000 mg/day or high blood concentrations above 70 μg/ml during pregnancy [6]. Combined use of antiepileptic drugs is also associated with a high risk of fetal malformations. The risk of major congenital malformations is influenced not only by the type of antiepileptic drug used but also by dose and other variables, which should be taken into account in the management of epilepsy in women of childbearing potential [4]. Thus, preconceptional counseling for a woman with epilepsy who is taking antiepileptic drugs is very important.

When a woman who is being treated with antiepileptic drugs, including VPA conceives, careful ultrasonographic observation of the fetus might be useful for predicting FVS. Witters *et al*. reported that prenatal nuchal edema could be the first presenting sign of FVS [7]. Because infants with FVS sometimes have multiple congenital heart diseases, this finding is reasonable and useful for predicting FVS. Contrary to our prediction, most reports of FVS have occurred in neonates and children, and we found only two reports that provided prenatal sonographic representations of FVS [7, 8] (Table 1). Witters *et al*. [7] reported an ultrasonographic feature of a fetal flat nasal bridge with a short nose at 21 weeks of gestation. The following pregnancy course was uneventful in their case, and the baby was born at term. The infant also had dysmorphic features seen in FVS: a long philtrum, thin upper lip, and long toes with a short fifth toe [7]. Kennelly and Moran [8] described a radial ray defect visualized by two- and three-dimensional prenatal ultrasonography at 19 weeks of gestation. The pregnancy was terminated, and the baby was revealed to have aortic valve stenosis postnatally [8]. In our case, contracture of the fetal wrist joint suggestive of a radial ray defect and saddle nose as a facial anomaly were detected by prenatal ultrasonography, indicating that the detection of these findings is a hallmark for precise prenatal diagnosis of FVS. In addition, the present case is the first case of FVS in which both contracture of the wrist joint and saddle nose were observed prenatally. In our case, fetal cardiac malformation, including ASD and VSD, was not detected prenatally because of a poor ultrasound image due to maternal obesity.

**Table 1:** Reported cases of fetal valproate syndrome detected by prenatal ultrasonography

Author	Maternal age	Antiepileptic drugs	US findings of fetus (gestational weeks)	Delivery	Additional postnatal findings
Witters *et al*. 2002 [7]	40 years	VPA; 1000 mg/day	Flat nasal bridge with short nose (21 weeks)	Term-delivery female	Long philtrum, thin upper lip, long toes
Kennelly and Moran 2007 [8]	N/A	VPA; dose N/A	Bilateral radial ray defects (19 weeks)	Termination of pregnancy	Aortic valve stenosis
Our case	27 years	VPA 1400 mg/day PB 140 mg/day CBZ 1200 mg/day	Contracture of right wrist joint suggestive of radial ray defect (25 weeks)	Term-delivery female	VSD, ASD, PDA; shallow philtrum; euryopia
			Saddle nose (37 weeks)		

*Abbreviations: US* Ultrasonography, *VPA* Valproate, *PB* Phenobarbital, *CBZ* Carbamazepine, *VSD* Ventricular septal defect, *ASD* Atrial septal defect, *PDA* Patent ductus arteriosus, *N/A* Not available

When radial ray defects and facial anomaly are observed by prenatal ultrasonography, differential diagnoses of FVS are trisomy 18, trisomy 13, Cornelia de Lange syndrome, Roberts syndrome, acrofacial dysostosis, Baller-Gerold syndrome, Fanconi anemia, Aase syndrome, thrombocytopenia-absent radius (TAR) syndrome, and Holt-Oram syndrome [8]. The facial anomaly in FVS is usually flat nasal bridge or saddle nose; facial anomalies seen in other syndromic disorders are micrognathia (trisomy 18, Cornelia de Lange syndrome, acrofacial dysostosis, Baller-Gerold syndrome, and TAR (thrombocytopenia with absent radius) syndrome, cleft lip and/or palate (trisomy 13, Roberts syndrome, and Aase syndrome), mandibular protrusion (Cornelia de Lange syndrome), microphthalmus (Fanconi anemia), and hypertelorism (Holt-Oram syndrome) [8]. VACTERL association (vertebral anomalies, anal atresia, cardiovascular anomalies, tracheoesophageal fistula, esophageal atresia, renal (kidney) and/or radial anomalies, limb defects) is associated with radial ray defects, but it is not usually accompanied by facial anomaly [8]. Although Binder syndrome is characterized by a flat midface and nasal hypoplasia, it is not usually accompanied by radial ray defects [9].

## Conclusions

The present case indicated the possibility of prenatal diagnosis of FVS using ultrasonography. Especially, detection of contracture of the fetal wrist joint and saddle nose seemed to have diagnostic value. When obstetricians manage women with epilepsy using epileptic drugs without preconceptional counseling, meticulous ultrasonographic observation should be performed to prevent serious morbidity and mortality in postnatal life. Furthermore, when multiple malformations to an extensive degree exist, early prenatal diagnosis may lead to more appropriate management of FVS, including termination of pregnancy and providing counseling for future pregnancies.
